# Transcriptomic Analysis of *Broussonetia papyrifera* Fruit Under Manganese Stress and Mining of Flavonoid Synthesis Genes

**DOI:** 10.3390/plants14060883

**Published:** 2025-03-12

**Authors:** Zhiyuan Hu, Yiwang Tang, Jihui Zhang, Taotao Li, Yihan Wang, Yani Huang, Yunlin Zhao, Guiyan Yang, Zhenggang Xu

**Affiliations:** 1 Hunan Provincial Key Lab of Dark Tea and Jin-Hua, School of Materials and Chemical Engineering, Hunan City University, Yiyang 413000, China; huzhiyuan@hncu.edu.cn (Z.H.); litaotao@hncu.edu.cn (T.L.); wangyihan870428@163.com (Y.W.); 2 College of Life and Environmental Sciences, Central South University of Forestry & Technology, Changsha 410004, China; tangyw2024@163.com (Y.T.); rssq198677@163.com (Y.Z.); 3 College of Forestry, Northwest A & F University, Yangling 712100, China; zhangjihui@nwafu.edu.cn (J.Z.); huangyani.358@nwafu.edu.cn (Y.H.); yangguiyan@nwafu.edu.cn (G.Y.)

**Keywords:** *Broussonetia papyrifera*, heavy metal stress, flavonoid, molecular response

## Abstract

*Broussonetia papyrifera* is a deciduous tree with significant economic and medicinal value. It demonstrates notable physiological adaptability to mining areas with severe manganese contamination and is a pioneering species in the field of ecological restoration. Flavonoids are vital secondary metabolites that improve plant resilience to environmental stresses. In the study presented herein, immature and mature fruits of *B. papyrifera* grown in normal and high manganese environments were used as the test materials. *B. papyrifera* fruit was subjected to transcriptome sequencing via high-throughput sequencing technology to analyze its flavonoid metabolic pathways and related genes. Transcriptome sequencing identified a total of 46,072 unigenes, with an average length of 1248 bp and a percentage of Q30 bases ranging from 92.45 to 93.17%. Furthermore, 31,792 unigenes (69% of the total) were annotated using eight databases, including the GO and KEGG. Analysis of KEGG metabolic pathways and flavonoid content trends in *B. papyrifera* fruits revealed four unigenes with strong links to the flavonoid biosynthesis pathway under manganese stress: flavone 3-hydroxylase, flavonoids 3′,5′-O-methyltransferase, chalcone synthase, and flavonol synthase. These unigenes may play important roles in regulating flavonoid synthesis in *B. papyrifera* fruits under manganese stress. This study lays the groundwork for functional gene research in *B. papyrifera*.

## 1. Introduction

Paper mulberry (*Broussonetia papyrifera*) is an ecologically, economically, and medicinally important tree species belonging to the family Moraceae [1,2]. Because of its long fibers and ease of preparation, this tree has been cultivated as a prime source of high-quality paper and is distributed worldwide [3]. In addition, *B. papyrifera* has been used to feed livestock for thousands of years owing to its high crude protein content and improved muscle quality [4]. The adaptability of *B. papyrifera* to the environment facilitates its colonization and expansion in local areas. *B. papyrifera* is a pioneer plant species in heavy metal-contaminated areas and is considered a candidate plant for phytoremediation [5,6,7]. *B. papyrifera* is a traditional Chinese medicine with positive effects on cardiovascular and neuropathic diseases [8]. The medicinal effects of *B. papyrifera* are mainly due to its rich secondary metabolites, of which flavonoids are the major functional components. To date, more than 70 flavonoids, including broussoflavone and kazinol, with antioxidant, anti-inflammatory, and antineoplastic activities have been identified in *B. papyrifera*, such as broussoflavone and kazinol [8,9]. Comparative genomic analysis has revealed an expansion in flavonoid biosynthetic gene families, accounting for the enhanced flavonoid biosynthesis in *B. papyrifera* [10].

Soil environmental pollution, particularly heavy metal toxicity, poses a major challenge to global ecosystem health. Heavy metals in soil are toxic to organisms and threaten human health through the food chain and other pathways [11]. Although *B. papyrifera* has potential as both a phytoremediator and livestock feed, its tendency to accumulate heavy metals in edible tissues raises significant concerns regarding food chain contamination. Field studies have demonstrated that manganese (Mn) accumulation in fruits derived from contaminated soils surpasses the safe consumption thresholds for livestock [12]. Therefore, further research is required to optimize these strategies for sustainable utilization. Faced with heavy metal stress, plants develop various defense strategies, with the synthesis of secondary metabolites being a key mechanism to regulate environmental stress, including phenolic compounds, sulfur-containing secondary metabolites, and nitrogen-containing secondary metabolites [13,14]. Under salt and drought stress, cotton exhibits elevated callose, chitinase, flavonoid, and phenol contents and higher secondary metabolism-related enzyme activities and transcript levels [15]. Plant secondary metabolites (PSMs) are not only regulatory substances for plants to cope with environmental stress but also the material basis for plants as food [16]. With rich secondary metabolites, *Gynostemma pentaphyllum* and *Houttuynia cordata* medicinal plants have been developed into healthy foods, such as Jiaosu [17]. Furthermore, PSMs are associated with abiotic stress tolerance, plant metabolite production, biostimulants, and functional foods [18].

Flavonoids are phenolic compounds that are important for plants to resist adverse environments and are functional substances. Flavonoid synthesis is an effective strategy against reactive oxygen species (ROS) [19]. Flavonoids may mediate ultraviolet protection in plants either by screening for harmful radiation or by minimizing the resulting oxidative stress [20]. Flavonoids play an important role in heavy metal tolerance [21]. Potent antioxidants from plants are of great interest as alternatives to synthetic antioxidants. In humans, flavonoids decrease organic hydroperoxide formation, induce antioxidant enzymes, such as superoxide dismutase and catalase, and inhibit enzymes that participate in oxidative processes [22]. In recent years, omics technology has been widely used to better understand the mechanisms underlying flavonoid synthesis [23]. Eight biosynthesis branches and four important intermediate metabolites have been identified [24]. Transcriptomics plays an important role in the analysis of key genes involved in flavonoid synthesis, and studies have shown that genes closely related to the flavonoid metabolism pathway are concentrated in v-myb avian myeloblastosis viral oncogene homolog (*MYB*), basic Helix-Loop-Helix (*Bhlh*), and tryptophan-aspartic acid 40 (*WD40*) families [24].

Currently, research on flavonoids in *B. papyrifera* has mainly focused on the leaves [25] and root bark [26], with researchers paying less attention to the fruits. Integrative metabolome and transcriptomic analyses of *B. papyrifera* leaves have identified several key genes regulating flavonoid accumulation, such as those encoding chalcone synthase (*CHS*), chalcone isomerase (*CHI*)*,* and dihydroflavonol-4-reductase (*DFR*) [27]. As a dioecious tree with globose syncarps, *B. papyrifera* is dispersed by birds and small mammals [28]. The fruit of *B. papyrifera* is rich in flavonoids and other secondary metabolites [12]. It is not only an important food source for animals but can also be used to make many kinds of food, such as juice. Although transcriptomic analysis has been used to analyze flavonoid synthesis and stress resistance response mechanisms in *B. papyrifera* leaves [29], few studies have reported on its application to fruits. To more effectively develop the fruit of *B. papyrifera* and understand its response to environmental stress, this study is the first attempt to use transcriptomic technology to explore the key genes regulating flavonoid synthesis in the fruit and establish the gene associations between flavonoid synthesis and stress resistance.

## 2. Results

### 2.1. The Relationship Between the Flavonoid Content of B. papyrifera Fruit and the Soil Environment

In this study, *B. papyrifera* fruits were collected from areas with and without manganese contamination. In each area, fruits were collected at two different development stages: immature and completely mature. Thus, the samples used in this study encompassed four different types: contaminated immature fruit (CIF), contaminated mature fruit (CMF), garden immature fruit (GIF), and garden mature fruit (GMF).

**Figure 1:**
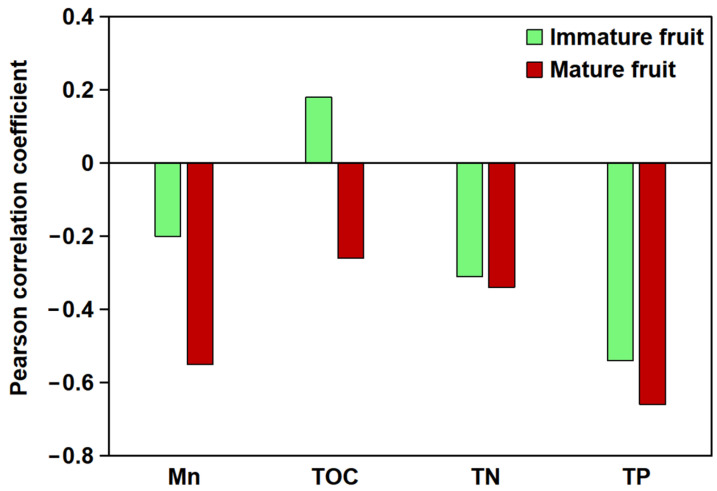
Pearson correlation analysis between soil elements (Mn, TOC, TN, and TP) and total flavonoid content in *B. papyrifera* fruits. The *X*-axis denotes various soil elements, while the *Y*-axis indicates the correlation coefficient.

The relationship between the total flavonoid content in *B. papyrifera* fruits and soil parameters at different developmental stages and soil carbon, nitrogen, phosphorus, and Mn content was analyzed (Figure 1). The results showed that the total flavonoid content of *B. papyrifera* fruits was highly correlated with soil Mn content. A negative correlation was observed between the total flavonoid content of immature fruits and soil Mn content (*p* > 0.05), whereas a significant negative correlation was evident between the flavonoid content of mature fruits and soil Mn content (*p* < 0.01). Furthermore, the effect of soil nutrient composition on the flavonoid content of *B. papyrifera* fruit was pronounced, with the following correlation strengths: total phosphorus (TP) > total nitrogen (TN) > total organic carbon (TOC).

### 2.2. Transcriptomic Data Analysis

Immature and mature fruits of *B. papyrifera* from the Mn mining and control areas were selected for transcriptome sequencing, and four cDNA libraries were constructed from the four samples. The sequencing results, as presented in Table 1, demonstrated that a total of 27.04 Gb of clean data were obtained, with CIF, CMF, GIF, and GMF yielding 21,185,851, 22,811,605, 24,414,219, and 22,202,511 clean reads, respectively. The percentage of Q30 bases was 92.45% or higher, and the GC content was above 47.02%. The raw sequencing reads exhibited an average length of 150 bp (paired-end), with 85.3% of the reads exceeding 100 bp. Sequencing revealed that immature and mature fruit samples of *B. papyrifera* were of high quality and suitable for analysis. A total of 46,072 unigenes were obtained from the four groups of samples following assembly, with an average length of 1248 bp. Of these, 26,394 unigenes were shorter than 900 bp, representing 57.29% of the total unigenes, and the longest was 17,072 bp (Figure S1). The high-quality assembly (N50 = 1248 bp) and Q30 results (>92.45%) further confirmed that minimal RNA degradation occurred during sample processing.

### 2.3. Annotation of Gene Functions

A comparative analysis of the 46,072 unigenes was performed using the Non-Redundant Protein Sequence Database (NR), Protein family (Pfam), euKaryotic Orthologous Groups (KOG), Clusters of Orthologous Groups (COG), Orthologous Groups of protein (eggNOG), a manually annotated and reviewed protein sequence database (Swiss-Prot), Kyoto Encyclopedia of Genes and Genomes (KEGG), and Gene Ontology (GO) databases. This resulted in the annotation of 31,792 unigenes in eight databases, representing 69% of the total (Table 2). The eggNOG and Pfam databases were the most heavily utilized, with 62.88% and 51.01% of the unigenes annotated in these databases, respectively.

The high annotation rate and sequencing quality indicated robust data integrity, providing a solid foundation for downstream comparative analysis. Leveraging this high-quality transcriptome dataset, we subsequently analyzed DEGs (differentially expressed genes) between the Mn-exposed and control groups.

### 2.4. Screening of DEGs

**Figure 2:**
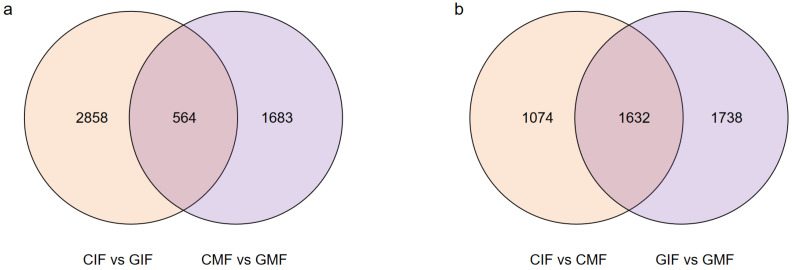
Pairwise comparison of DEGs between the sample groups. (**a**) CIF vs. GIF and CMF vs. GMF. (**b**) CIF vs. CMF and GIF vs. GMF. Venn diagrams display overlaps of DEGs between comparisons.

DEGs were screened based on the criteria of False Discovery Rate (FDR) < 0.01 and Fold Change (FC) ≥ 2, and pairwise comparisons of DEGs were conducted for immature and mature fruits from Mn mining and control areas. The comparison between CIF and GIF revealed a total of 3422 DEGs (Figure 2a), including 2168 upregulated and 1254 downregulated DEGs. The comparison between CMF and GMF revealed 2247 DEGs (Figure 2a), including 944 upregulated and 1303 downregulated DEGs. The comparison between CIF and CMF revealed 2706 DEGs (Figure 2b), including 1670 upregulated and 1036 downregulated DEGs. Similarly, the comparison between GIF and GMF revealed 3370 DEGs (Figure 2b), including 2055 upregulated and 1315 downregulated DEGs.

Among the DEGs, some were identified in the comparisons between CIF and GIF and CMF and GMF. These included 345 upregulated and 219 downregulated genes. Similarly, some were in the comparisons between CIF and CMF and GIF and GMF. These included 1081 upregulated and 551 downregulated genes. The DEGs were functionally annotated using the following eight databases: NR, SwissProt, KEGG, COG, KOG, Egg-NOG, GO, and Pfam. The results are summarized in Table 3. Among the comparisons between CIF and GIF, CMF and GMF, CIF and CMF, and GIF and GMF, 2989, 2053, 2451, and 3016 DEGs were annotated in the eight databases, respectively.

### 2.5. RT-qPCR Validation of DEGs

**Figure 3:**
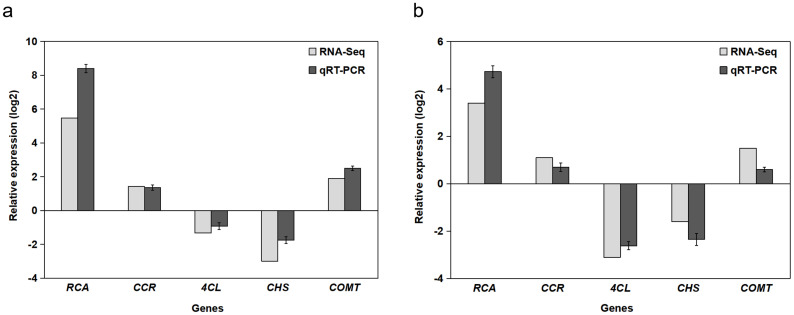
Validation of transcriptome data using RT-qPCR for five randomly selected genes. (**a**) Immature fruit and (**b**) mature fruit. “Relative expression log2” on the *Y*-axis represents log2-transformed fold changes normalized to the reference gene *Actin* and calibrated against the respective control group. Error bars indicate the standard deviation (*n* = 3 biological replicates).

Five genes were randomly selected for transcriptome sequencing and expression level verification using RT-qPCR. Figure 3a illustrates the expression of these five genes in the immature fruit of *B. papyrifera*, and Figure 3b depicts their expression in mature fruit. The results demonstrated that the expression levels of the same gene under both Illumina sequencing and RT-qPCR were largely consistent. This suggests that the transcriptome sequencing data in this study are accurate and reliable and can be utilized for further analysis.

### 2.6. GO Annotation of DEGs

**Figure 4:**
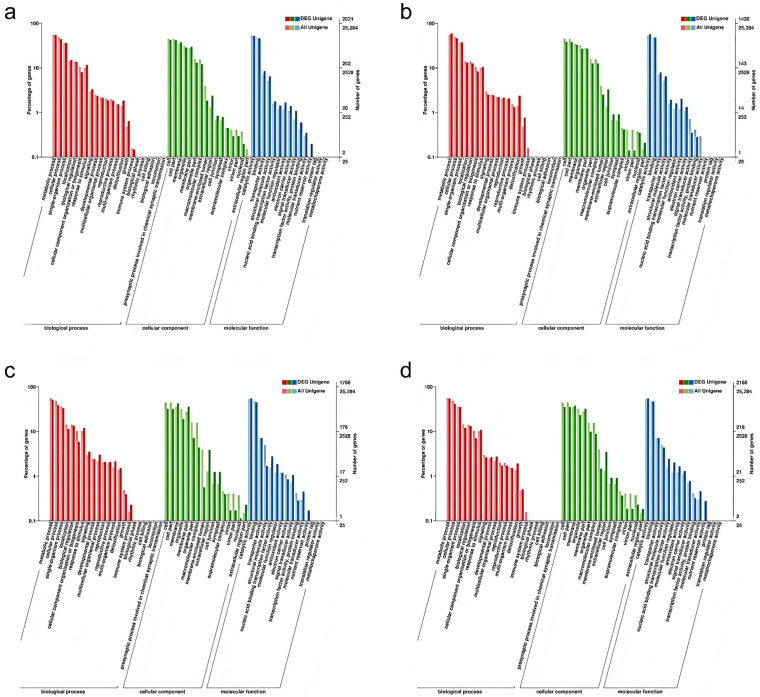
GO enrichment analysis of DEGs in three categories: biological process, cellular component, and molecular function. (**a**) CIF vs. GIF. (**b**) CMF vs. GMF. (**c**) CIF vs. CMF. (**d**) GIF vs. GMF. The most significantly enriched terms for each category are presented. Bar length represents the number of DEGs assigned to each term.

To provide a comprehensive description of the functional attributes of the DEGs, a GO systematic functional classification of the DEGs was conducted. A total of 2021 DEGs were annotated to the GO database for CIF vs. GIF (Figure 4a), of which 1647 were annotated to molecular functions, 1358 were annotated to cellular components, and 1499 were annotated to biological processes. In the CMF vs. GMF comparison, 1438 DEGs were annotated in the GO database (Figure 4b), of which 1217 were annotated to molecular functions, 885 were annotated to cellular components, and 1113 were annotated to biological processes. A total of 1766 DEGs were annotated in the GO database for CIF vs. CMF (Figure 4c), of which 1413 were annotated to molecular functions, 1112 were annotated to cellular components, and 1274 were annotated to biological processes. In total, 2168 DEGs were annotated in the GO databases for GIF and GMF (Figure 4d), of which 1799 were annotated as molecular function, 1364 were annotated as cellular components, and 1617 were annotated as biological processes.

These three groupings can be subdivided independently into different functional subterms, each corresponding to an attribute. As shown in Figure 4, the differential genes of CIF vs. GIF, CMF vs. GMF, CIF vs. CMF, and GIF vs. GMF were enriched in 48, 42, 45, and 45 different taxa, respectively, in which metabolic processes, cellular processes, and single-organism processes were dominant in the biological process; cell parts and cells were most significantly enriched in cellular composition; and catalytic activity and binding were the dominant taxa in molecular function.

### 2.7. KEGG Pathway Analysis of DEGs

**Figure 5:**
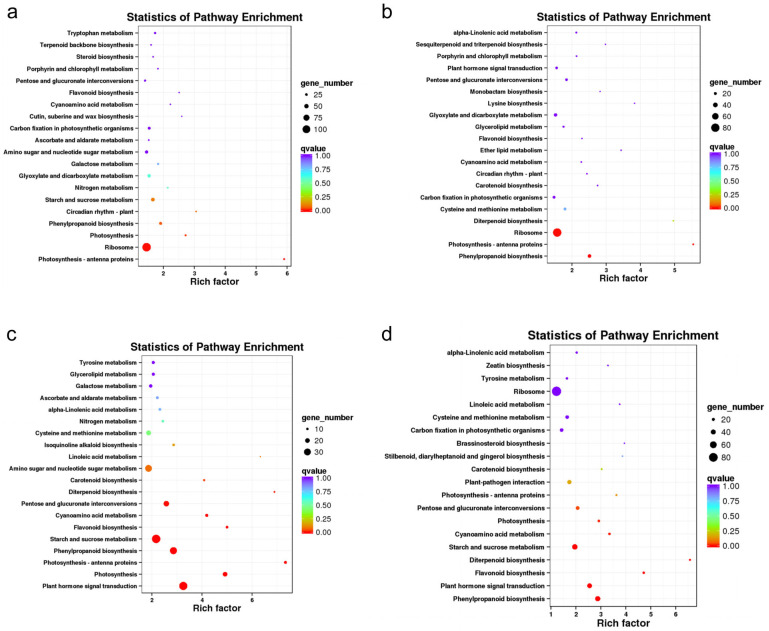
KEGG pathway enrichment analysis of DEGs. (**a**) CIF vs. GIF. (**b**) CMF vs. GMF. (**c**) CIF vs. CMF. (**d**) GIF vs. GMF. The top 20 enriched pathways are ranked by *p*-value. Circle size indicates the number of DEGs in each pathway; color intensity represents the enrichment significance.

KEGG pathway analysis showed that 1128, 750, 828, and 1069 DEGs were annotated to KEGG pathways in the CIF vs. GIF, CMF vs. GMF, CIF vs. CMF, and GIF vs. GMF comparisons, respectively; these DEGs were annotated to 113, 102, 109, and 115 KEGG pathways, respectively. Figure 5 shows the 20 most significantly enriched KEGG pathways in the four comparison groups. In the comparison between CIF and GIF (Figure 5a), the three most significantly enriched KEGG pathways were photosynthesis–antenna proteins, ribosomes, and photosynthesis pathways. Similarly, in the comparison between CMF and GMF (Figure 5b), the top three pathways were phenylpropanoid biosynthesis, photosynthesis–antenna proteins, and ribosomes. In the comparison between CIF and CMF (Figure 5c), the top three KEGG pathways were plant hormone signal transduction, photosynthesis, and photosynthesis–antenna proteins. In the comparison between GIF and GMF (Figure 5d), the top three KEGG pathways were phenylpropanoid biosynthesis, plant hormone signal transduction, and flavonoid biosynthesis.

### 2.8. Analysis of DEGs Related to Flavonoid Synthesis

Analysis of the significantly enriched DEGs in the flavonoid biosynthesis pathway (Table 4) revealed that in the comparison between CIF and GIF, the DEGs *CYP* and *DFR* were upregulated while *F3H* and *FAOMT* were downregulated; in the comparison between CMF and GMF, *CYP* was upregulated while *CHS* and *FLS* were downregulated; in the comparison between CIF and CMF, *BAHD*, *CCoAOMT*, *CHI*, *F3H*, *FLS*, *ANR*, and *CYP* were upregulated, and in the comparison between GIF and GMF, *CHI*, *CCoAOMT*, *DFR*, and *FLS* were upregulated.

## 3. Discussion

Our transcriptomic and biochemical analyses collectively revealed that Mn stress reprograms flavonoid metabolism in *B. papyrifera* fruits, which has both ecological and economic ramifications. The dual regulatory role of Mn in plant flavonoid metabolism, in which biosynthesis is facilitated at low concentrations and metabolic homeostasis is disrupted at elevated levels, is prominently exemplified in *B. papyrifera* fruits. Under optimal Mn availability, flavonoid synthesis is enhanced through the function of Mn as a cofactor for pivotal enzymes, including phenylalanine ammonia-lyase (*PAL*) and *CHS*, which drive the phenylpropanoid pathway [30,31,32]. In contrast, excessive Mn accumulation in mining-exposed *B. papyrifera* fruits induces systemic toxicity, which is characterized by oxidative stress, impaired stomatal conductance (reducing CO_2_ assimilation), and antagonistic interactions with essential micronutrients (e.g., Fe and Mg) [33,34,35,36]. These physiological perturbations are directly correlated with the downregulation of core flavonoid biosynthetic genes (*F3H*, *CHS*, *FAOMT*, and *FLS*), mirroring the Mn toxicity patterns observed in litchi (pericarp darkening) [35] and grape (oxidative suppression of *PAL*/*CHS*) [31]. Notably, the inhibition of *F3H* and *CHS* aligns with diminished flavonoid content, suggesting that Mn overload disrupts carbon allocation to flavonoid precursors (e.g., naringenin) and compromises the synthesis of stress-mitigating flavonols [37,38]. These factors likely contribute to the reduced flavonoid content observed in *B. papyrifera* fruits from Mn mining areas.

Transcriptomic profiling of *B. papyrifera* fruit revealed a multifaceted adaptive strategy to Mn stress. DEGs were enriched in biological processes such as detoxification, antioxidant response, and transcription factor activity, indicating a concerted effort to counteract Mn-induced oxidative damage and restore metabolic equilibrium. For instance, novel compounds have been detected in *B. papyrifera* branches, and these compounds can inhibit ROS production in THP-1 cells [39]. Furthermore, *B. papyrifera* has been shown to maintain ROS homeostasis through symbiosis with arbuscular mycorrhizal fungi, a process that enhances catalase (CAT), peroxidase (POD), and superoxide dismutase (SOD) activities in the roots [40]. This reprogramming starkly contrasts with the maturation-associated flavonoid dynamics in the control fruits, where *CHI* and *DFR* upregulation drives flavonoid diversification, underscoring the plasticity of the pathway under developmental versus environmental cues. The observation that flavonoid-related genes are suppressed in a conserved manner across Mn-stressed species, including *B. papyrifera*, litchi, and grape, suggests a shared ROS-mediated inhibition mechanism [35,36]. In *B. papyrifera*, the reduced activity of 2-oxoglutarate-dependent dioxygenases (e.g., *F3H* and *FLS*) under excess Mn may reflect competitive inhibition by Mn^2+^ substitution for Fe^2+^ in enzyme active sites [41], a hypothesis that requires validation through kinetic assays. Furthermore, Mn-induced dysregulation of *FAOMT*, which methylates flavonoid glycosides at the 3′-OH and 5′-OH positions to promote generation [42], could accelerate flavonoid degradation in fruits from mining areas, thereby compounding the loss of bioactive metabolites.

By synthesizing phenotypic and molecular data, this study has established a mechanistic framework linking Mn toxicity to flavonoid metabolism in *B. papyrifera*. The suppression of structural genes (*CHS* and *F3H*) and auxiliary regulators (*FAOMT* and *FLS*) not only reduces flavonoid diversity but also weakens the plant’s capacity to mitigate oxidative stress, thus creating a feedback loop that exacerbates Mn toxicity. These insights align with the broader patterns of Mn phytotoxicity in non-hyperaccumulator species, where disrupted micronutrient homeostasis and enzyme dysfunction converge to impair specialized metabolism [34,43,44,45]. Future studies should prioritize the functional characterization of candidate DEGs (e.g., *F3H*, *FAOMT*, *CHS*, and *FLS*) to elucidate their roles in Mn detoxification and flavonoid regulation, particularly in perennial species adapted to metalliferous environments. Notably, the comparison between immature and mature control fruits (GIF vs. GMF) revealed that *CHI*, *CCoAOMT*, *DFR*, and *FLS* were upregulated, suggesting that fruit maturation itself could also drive flavonoid pathway activation.

In addition to elucidating molecular responses, our findings have practical implications for phytoremediation. *B. papyrifera*’s ability to thrive in Mn-contaminated soils [5] and modulate flavonoid synthesis under stress positions it as a dual-purpose candidate capable of (1) stabilizing heavy metals via rhizosphere interactions [7] and (2) producing value-added metabolites for biorefinery. For example, Mn-stressed fruits, despite having reduced flavonoid content, may still serve as raw materials for antioxidant extracts, whereas high-biomass foliage aids in soil remediation. Future field trials should evaluate the tradeoffs between Mn accumulation and metabolite yields to optimize phytomanagement strategies. Modulation of flavonoid synthesis genes under Mn stress also leads to functional food development. *B. papyrifera* fruits are traditionally used in fermented beverages, such as Jiaosu [17], where flavonoids contribute to its antioxidant and anti-inflammatory properties. Our identification of *FLS* and *FAOMT* as Mn-responsive genes suggests that soil Mn levels could be strategically managed to enhance specific flavonoid subclasses (e.g., flavonols or methylated derivatives) in cultivated populations. Such targeted cultivation along with postharvest processing (e.g., microbial fermentation to hydrolyze glycosides) may maximize the nutraceutical potential of these fruits in heavy metal-affected regions. In addition, the dual role of *B. papyrifera* as a phytoremediator and fodder source necessitates careful risk–benefit analysis. It is imperative to implement strategies aimed at mitigating the associated risks. For example, farmers in heavy metal-polluted regions often reserve *B. papyrifera* biomass for non-feed purposes, such as bioenergy production [46], and use uncontaminated plantations for fodder. Additionally, agronomic intervention measures, such as co-cultivation with metal-immobilizing plants (e.g., *Pennisetum purpureum* Schum and *Melia azedarach linnaeus*), can suppress metal uptake by *B. papyrifera* without hindering its phytoremediation efficacy, further mitigating contamination risks [6].

## 4. Materials and Methods

### 4.1. Plant Materials and Soil Analysis

*B. papyrifera* is found in various habitats. In this study, we selected wild-type *B. papyrifera* populations growing naturally in the Xiangtan manganese mining area (27°53′ N, 112°45′ E) and the campus of Central South Forestry University (28°08′ N, 113°00′ E) as the study material. The distance between the two sites was approximately 25 km, and the climatic conditions were the same. The Xiangtan manganese mine was once an important mining area, and manganese, cadmium, and other metals in the soil significantly exceeded the standards [47]. *B. papyrifera* is a pioneering plant in the Xiangtan manganese mine. Compared to the campus, *B. papyrifera* in the Xiangtan manganese mine faces severe heavy metal stress [12]. The fruits of *B. papyrifera* have a long maturation process, and fruits at different developmental stages would coexist on the same tree. In July 2019 and September 2019, we collected CIF and CMF of *B. papyrifera* from the Xiangtan manganese mining area, respectively, and collected GIF and GMF of *B. papyrifera* on campus at the same stage as the controls. Freshly harvested fruits were immediately cut into small pieces (approximately 0.5 cm) and flash-frozen in liquid nitrogen for 5 min to preserve RNA integrity. The frozen samples were then transferred to a −80 °C freezer for long-term storage until RNA extraction. Additionally, soil samples were taken from the 0 to 20 cm layer within a 50 cm radius centered on the sampled *B. papyrifera* trunk. The samples were air-dried, ground, and sieved using a 100-mesh sieve for subsequent analyses.

The soil organic carbon content was determined using spectrophotometry, total nitrogen content was determined using the Kjeldahl method, total phosphorus content was determined using the molybdenum–antimony anti-colorimetric method, and Mn content was determined using flame atomic absorption spectroscopy. The total flavonoid content of *B. papyrifera* fruit was determined using the aluminum ion complexation method. For further details on the testing of the plant and soil samples, please refer to our previous report [12].

### 4.2. RNA Extraction, Library Construction, and Sequencing

Total RNA was extracted from each sample using an EASYspin Plus Plant RNA Rapid Extraction Kit (Aidlab Biotech, Beijing, China). The extracted RNA was subjected to 1.0% agarose gel electrophoresis to detect potential degradation or contamination [48]. RNA purity was determined using a nucleic acid protein analyzer spectrophotometer (Implen, Westlake Village, CA, USA) [49], and RNA concentration and integrity were analyzed using the Qubit RNA Assay Kit (Life Technologies, Carlsbad, CA, USA) and RNA Nano 6000 Assay Kit (Agilent Technologies, Santa Clara, CA, USA) [50].

### 4.3. Data Assembly and Gene Function Annotation

Raw RNA-seq reads were subjected to quality control using FastQC [51] to assess sequence quality, GC content, and potential adapter contamination. Low-quality reads (Phred score < 20), adapter sequences, and reads shorter than 50 bp were trimmed using Trimmomatic [52]. rRNA contamination was identified and removed using SortMeRNA [53] against the SILVA rRNA database using SortMeRNA. High-quality reads were assembled de novo using Trinity [54] with the default parameters (k-mer size = 25, min_contig_length = 200). Redundant transcripts were clustered using CD-HIT-EST [55] at a 95% sequence identity threshold to generate a non-redundant unigene set. The accuracy of the assembled transcripts was validated by remapping raw reads in the final assembly using Bowtie2 [56]. Transcript abundance was quantified via Salmon [57] in alignment-based mode. Potential misassemblies were identified and rectified using Pilon [58] with iterative polishing.

Open reading frames were predicted using TransDecoder [59] with a minimum length of 100 amino acids. Following this, the unigenes were compared to the NR, Pfam, KOG, COG, eggNOG, Swiss-Prot (manually annotated and reviewed protein sequence database), KEGG, and GO. Functional classification of the unigenes in KEGG was performed using KOBAS [60]. Following the prediction of the amino acid sequences of the unigenes, HMMER software (v3.3.2) was employed for comparisons with the Pfam database to obtain annotation information for the unigenes [61].

### 4.4. Quantitative Gene Expression Level and Differential Expression Analysis

Bowtie was used to align the sequenced reads with the unigene library [62]. Because RSEM (https://github.com/deweylab/RSEM, accessed on 9 March 2025) is compatible with de novo transcriptomes and does not require a reference genome [63], it has high reliability among similar software [64]. Therefore, RSEM was used to quantify the gene expression levels in this study. First, the reference transcripts were preprocessed using the scripts rsem-prepare-reference with genome annotations or de novo assemblies. Second, rsem-calculate-expression aligns reads and employs an expectation–maximization (EM) algorithm to estimate transcript abundance and probabilistically resolve ambiguous reads. Bayesian Gibbs sampling was employed to compute 95% credibility intervals and posterior mean estimates. The expression abundance of each unigene was represented as Fragments Per Kilobase of transcript per Million mapped reads (FPKM).

Differentially expressed genes (DEGs) between two samples were analyzed using EBSeq [65]. Significant *p*-values obtained from the original hypothesis test were corrected using the Benjamini–Hochberg method, and the final corrected *p*-value, that is, the False Discovery Rate (FDR), was used as the key indicator for DEG screening. In the screening process, FDR < 0.01 and differential multiple fold change (FC) ≥ 2 were used as the screening criteria [66].

### 4.5. RT-qPCR to Verify DEGs

The expression levels of the DEGs in the samples were validated using quantitative real-time polymerase chain reaction (RT-qPCR) [67]. Total RNA was extracted from the samples using the CTAB method, and reverse transcription amplification was performed using a Goldenstar RT6 cDNA Synthesis Kit Ver 2 (Tsingke Biotech, Beijing, China). The RNA template, gDNA remover, 10 × gDNA remover buffer, and RNase-free water were combined and incubated at 42 °C for 2 min, followed by incubation at 60 °C for 5 min. The mixture was then rapidly cooled. Following brief centrifugation, dNTP Mix (1 µL), Randomer primer (1 µL), 5 × Goldenstar TM Buffer (4 µL), DTT (2 M) (1 µL), and Goldenstar TM RT6 enzyme (a volume of 1 µL) were combined with 2 µL of RNase-free water, and the mixture was incubated at 25 °C for 10 min, 50 °C for 30 min, and 85 °C for 5 min. The cDNA obtained through reverse transcription was diluted and used as a template for RT-qPCR. Considering that the *Actin* gene of *B. papyrifera* is a stable reference gene for RT-PCR [2,68], it was selected as an internal reference for PCR amplification. RT-qPCR was performed using an FQD-96A fluorescence quantitative PCR instrument (Tsingke Biotech, Beijing, China). The primers are shown in Table S1. The components of the amplification system were as follows: 2 × T5 Fast qPCR Mix (10 μL), 10 μM Primer F (0.8 μL), 10 μM Primer F (0.8 μL), cDNA Template (1 μL), and ddH_2_O (7.4 μL), a total of 20 µL. The specific amplification steps are shown in Table S2. Relative expression levels were calculated using the 2^−ΔΔCT^ method [69]. Briefly, the Ct values of target genes were normalized to BpACT2, and fold changes were derived by comparing the ΔCt values between the Mn-exposed and control groups. The final expression values were log2-transformed for visualization.

### 4.6. Statistical Analysis

The experimental data were statistically analyzed using SPSS 23 software. Pearson’s coefficient was used to analyze the correlation between the flavonoid content of *B. papyrifera* fruit and the soil element content [70]. Standard deviations were used to represent the variability of the samples. Differences were considered statistically significant at *p* < 0.05.

## 5. Conclusions

Transcriptomic analysis of *B. papyrifera* fruits from Mn mining and control areas was performed. The results demonstrated that in the context of disparate Mn environments, the DEGs in *B. papyrifera* fruits were predominantly associated with functions such as response to stimuli, detoxification, growth, signaling, and transcription factor activity. Furthermore, examination of the DEGs involved in the flavonoid synthesis pathway revealed that *F3H*, *FAOMT*, *CHS*, and *FLS* may be pivotal genes influencing flavonoid synthesis in the fruit of *B. papyrifera*. Further research is required to elucidate the effect of Mn on the specific mechanism of flavonoid synthesis and to ascertain how the content and quality of flavonoids in plants can be enhanced by regulating the Mn content in the soil. Additionally, the potential of genetic engineering techniques to develop new plant varieties with higher flavonoid content should be considered to meet the demand for healthy foods.

## Figures and Tables

**Table 1:** RNA sequencing results of *B. papyrifera* fruit.

Sample	Read Number	Base Number	GC Content	%≥Q30
CIF	21,185,851	6,325,128,368	47.37%	92.77%
GIF	22,811,605	6,813,841,942	47.02%	92.45%
CMF	24,414,219	7,279,736,050	48.12%	93.17%
GMF	22,202,511	6,625,058,020	47.88%	92.88%

Read Number: Total number of pair-end reads in clean data; Base Number: Total number of bases in clean data; GC Content: GC content in clean data; %≥Q30: Percentage of bases with a quality value greater than or equal to 30 in clean data. CIF: contaminated immature fruit; CMF: contaminated mature fruit; GIF: garden immature fruit; GMF: garden mature fruit.

**Table 2:** Annotation results of *B. papyrifera* fruit unigenes in the different databases.

Database	Number of Unigenes	Percentage (%)
GO	21,389	46.43
KEGG	10,048	21.81
KOG	16,670	36.18
Pfam	23,501	51.01
Swiss-Prot	18,854	40.92
NR	14,275	30.98
COG	12,044	26.14
Egg-NOG	28,972	62.88
Total unigenes	46,072	100

Note: KEGG, Kyoto Encyclopedia of Genes and Genomes; GO, Gene Ontology.

**Table 3:** Functional annotation of DEGs in the eight databases.

DEG Set	Annotated	COG	GO	KEGG	KOG	Pfam	nr	Swiss-Prot	eggNOG
CIF vs. GIF	2989	1388	2021	1128	1637	2427	2923	2047	2775
CMF vs. GMF	2053	941	1438	750	1025	1661	2042	1413	1934
CIF vs. CMF	2451	991	1766	828	1126	1954	2441	1853	2308
GIF vs. GMF	3016	1314	2168	1069	1446	2455	2978	2232	2851

**Table 4:** DEGs related to flavonoid biosynthesis.

Grouping	Tendency	Genes	Name	Ko ID
CIF vs. GIF	Up	Cytochrome P450	*CYP*	K05280
	Up	Dihydroflavonol/flavanone 4-reductase	*DFR*	K13082
	Down	Flavone 3-hydroxylase	*F3H*	K00475
	Down	Flavonoid 3′,5′-O-methyltransferase	*FAOMT*	K00588
CMF vs. GMF	Up	Cytochrome P450	*CYP*	K00487
	Down	Chalcone synthase	*CHS*	K00660
	Down	Flavonol synthase	*FLS*	K05278
CIF vs. CMF	Up	BAHD acyltransferase	*BAHD*	K13065
	Up	Cytochrome P450	*CYP*	K00487
	Up	Caffeoyl-CoA-O-methyltransferase	*CCoAOMT*	K00588
	Up	Chalcone isomerase	*CHI*	K01859
	Up	Flavone 3-hydroxylase	*F3H*	K00475
	Up	Flavonol synthase	*FLS*	K05278
	Up	Anthocyanidin reductase	*ANR*	K08695
GIF vs. GMF	Up	Chalcone isomerase	*CHI*	K01859
	Up	Caffeoyl-CoA-O-methyltransferase	*CCoAOMT*	K00588
	Up	Dihydroflavonol/flavanone 4-reductase	*DFR*	K13082
	Up	Flavonol synthase	*FLS*	K05278

## Data Availability

The transcriptome data of *B. papyrifera* fruit have been uploaded to the China National Center for Bioinformation (https://www.cncb.ac.cn/) under accession number PRJCA011809. Other data are contained within the article and Supplementary Materials.

## Supplementary Materials

The following supporting information can be downloaded at: https://www.mdpi.com/article/10.3390/plants14060883/s1. Figure S1. Length distribution of unigenes in the *B. papyrifera* fruit transcriptome. The X-axis represents the length intervals of unigenes, and the Y-axis represents the number of unigenes of different lengths. Table S1. Primers used for RT-qPCR analysis. Table S2. PCR amplification system components and amplification steps.
