# Supplementary material for: Transcriptomic Analysis of Broussonetia papyrifera Fruit Under Manganese Stress and Mining of Flavonoid Synthesis Genes
Source: Plants (Basel). 2025 Mar 12;14(6):883. doi: 10.3390/plants14060883 (PMC11944339; doi:10.3390/plants14060883)
Supplement: Supplementary file 1 [file plants-14-00883-s001.zip › Table S1.pdf]

Table S1. Primers used for RT-qPCR analysis.

Gene name	Forward primer	Reverse primer
Actin	TACGCATTGAAGACCCTCCAC	TGGCCACACTTGCTTAGACAA
COMT	AAGCTACAAACGCAACAGACAAA	GCTGTGAAGAACGACTTTGCG
CHS	GACACAGTTAGGCGGAGTTGC	GAATCATCCTTCCATTTCTACCAG
4CL	AAGGGTACTACAACAATCCACAGG	ATGAGCTCTTTAATTCGGTCCAC
CCR	CAGTTGGCTGAAGAAGTGGTCC	TGGACGAGTCTTGTTGGAGTGA
RCA	TTCCCTGACCTTTGCCTCC	GTTTCTACATCGCCCCTGCTT
