# Supplementary material for: Transcriptomic Analysis of Broussonetia papyrifera Fruit Under Manganese Stress and Mining of Flavonoid Synthesis Genes
Source: Plants (Basel). 2025 Mar 12;14(6):883. doi: 10.3390/plants14060883 (PMC11944339; doi:10.3390/plants14060883)
Supplement: Supplementary file 1 [file plants-14-00883-s001.zip › Table S2.pdf]

Table S2. PCR amplification system components and amplification steps.

**Stage**	**Cycle number**	**Operating method**
1	1	95°C for 1 min
2	40	95°C for 15 s → 60°C for 15 s → 72°C for 30 s → read fluorescence
3	1	95°C for 5 s → 60°C for 1 min → warming up to 95°C at 0.11°C/s, during which fluorescence was read continuously (5 times for every 1°C of warming) → 50°C for 30s
