# Supplementary figures and images for: Transcriptomic Analysis of Broussonetia papyrifera Fruit Under Manganese Stress and Mining of Flavonoid Synthesis Genes
Source: Plants (Basel). 2025 Mar 12;14(6):883. doi: 10.3390/plants14060883 (PMC11944339; doi:10.3390/plants14060883)

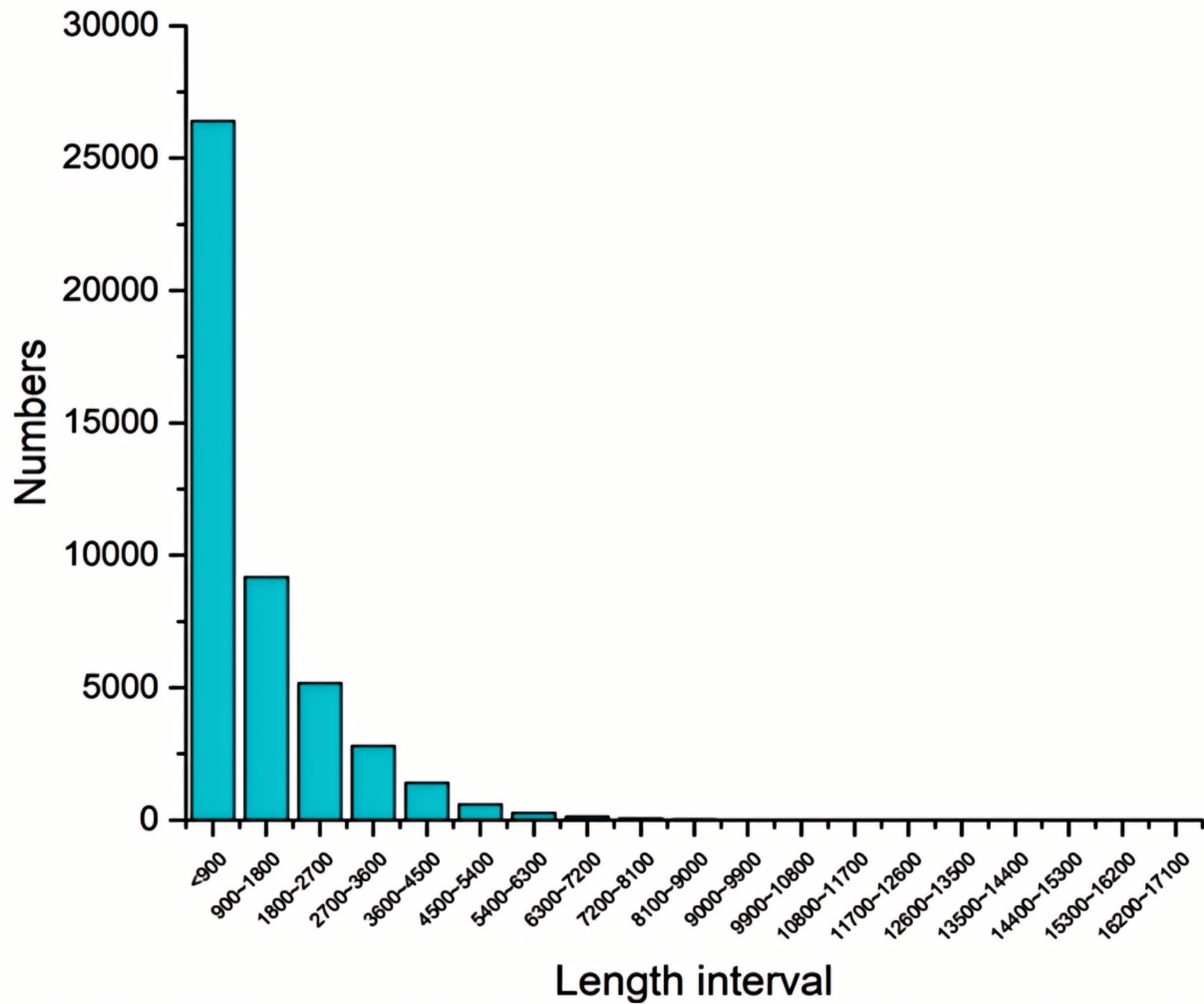

Supplement: Supplementary file 1 [file plants-14-00883-s001.zip › Figure S1.pdf]
